# *Sedum sarmentosum* Total Flavonoids Alleviate Schistosomiasis-Induced Liver Fibrosis by Altering TGF-*β*1 and Smad7 Expression

**DOI:** 10.1155/2020/2083697

**Published:** 2020-11-26

**Authors:** Peng-chun Yang, Wei-zhe Bai, Jing Wang, Cai-hua Yan, Wei-feng Huang, Shi-zhu Jiang

**Affiliations:** ^1^Department of Digestive Medicine, Affiliated Renhe Hospital of Three Gorges University, Yichang 443001, China; ^2^The Fifth Affiliated Hospital Sun Yat-sen University, Zhuhai 519000, China; ^3^The Institute of Infection and Inflammation, Department of Microbiology and Immunology, Medical College, China Three Gorges University, Yi Chang, Hubei 443002, China

## Abstract

**Objectives:**

Schistosomiasis is a parasitic disease that affects over 142 million people worldwide. The main causes of death of schistosomiasis include liver granuloma and secondary hepatic cirrhosis resulting from severe fibrosis. Despite intensive research, controlling liver fibrosis associated with schistosomiasis remains challenging. *Sedum sarmentosum* total flavonoid (SSTF) is a promising agent to reduce liver fibrosis with an unknown mechanism. Thus, the objectives of this study are to validate its effect on the liver fibrosis caused by schistosomiasis and to explore the underlying molecular mechanism.

**Methods:**

Sixty male Sprague-Dawley rats were randomly divided into six groups: one group of normal control and five groups of liver fibrosis induced by schistosomiasis japonica with or without SSTF or colchicine treatment, the latter serving as the positive control. Liver tissues from each animal were harvested to observe the degree and grade of hepatic fibrosis. We also measured the expression of transforming growth factor-beta 1 (TGF-*β*1) and Smad7 using RT-qPCR, Western blot, and immunohistochemistry.

**Results:**

Compared with the untreated model group, groups treated with SSTF at all three tested doses had significantly reduced hepatic fibrosis (*P* < 0.05). Each dose of SSTF also significantly reduced TGF-*β*1 protein expression and mRNA levels in the liver tissues (*P* < 0.05). In contrast, the middle and high doses of SSTF significantly increased Smad7 protein expression and mRNA levels (*P* < 0.05). Immunohistochemistry showed that each dose of SSTF reduced TGF-*β*1 protein expression (*P* < 0.05).

**Conclusion:**

Our results demonstrated that SSTF alleviated schistosomiasis japonica-induced hepatic fibrosis by inhibiting the TGF-*β*1/Smad7 pathway.

## 1. Introduction

Despite great efforts and major achievements, schistosomiasis remains an important global health concern [1, 2]. Circumoval granulomas and hepatic fibrosis are the major pathologic manifestations of schistosomiasis [3]. Currently, the only available treatment is praziquantel (PZQ), which can reduce fibrosis but cannot prevent reinfection [4].

Although the mechanism of non-schistosomiasis hepatic fibrosis is relatively clear, the mechanism of schistosomiasis-induced hepatic fibrosis remains elusive. Hepatic fibrosis is a pathophysiological process that involves an abnormal proliferation of connective tissue cells in the liver caused by various pathogenic factors. This results in excessive accumulation of extracellular matrix (ECM) components in the liver tissue. Hepatic fibrosis can progress to hepatic cirrhosis over time. The main source of ECM in the liver is the hepatic stellate cell (HSC), and activation of HSCs is the central link in the formation of hepatic fibrosis [5]. Therefore, investigating HSC activation is of great practical significance for exploring effective treatment strategies that target the underlying mechanism of hepatic fibrosis in schistosomiasis.

HSC activation is mainly mediated by some cytokines and their signal transduction pathways. Among these cytokines, the most important is transforming growth factor-beta 1 (TGF-*β*1) [6, 7]. The TGF-*β*1/Smads signaling pathway significantly influences HSC activation and ECM production. Smad7 is an important component of the TGF-*β*1/Smads signaling pathway. Smad7 is a negative regulator, and its overexpression can block HSC activation and hepatic fibrosis progression and even reverse hepatic fibrosis [8].

*Sedum sarmentosum* total flavanones (SSTF) are a mixture of flavonoid compounds extracted from chuipencao in traditional Chinese herbal medicine. SSTF contains quercetin, kaempferin, luteolin, and hyperin [9, 10] and has antibacterial, anti-inflammatory, and antioxidant properties. It has been demonstrated that SSTF can promote liver cell regeneration, induce HSC apoptosis, and prevent fibrosis after liver or kidney injury [11–14]. Thus, SSTF has a variety of pharmacological effects that could be targeted to treat inflammatory diseases, such as hepatitis [12]. However, the pharmacological effects of SSTF on fibrosis remain unclear.

In this study, we evaluated the therapeutic effects of SSTF against schistosomiasis-induced hepatic fibrosis and further sought to explore the potential mechanism of its therapeutic effect. We hypothesized that SSTF exerts its antifibrosis effect through inhibiting the TGF-*β*1/Smad7 signaling pathway. To test our hypothesis, we observed the effect of three different doses of SSTF on protein expression and mRNA levels of TGF-*β*1 and Smad7 using a rat model of schistosomiasis-induced liver fibrosis.

## 2. Materials and Methods

### 2.1. Animals

Sixty male Sprague-Dawley (SD) rats weighing 240 ± 20 g (license number: SCXK (E) 2015-0018, grade: SPF) were purchased from the Animal Laboratory SPF Center of Huazhong University of Science and Technology, Wuhan, Hubei Province, and raised in the SPF animal laboratory of the Experimental Animal Center of the Three Gorges University in Yichang City, Hubei Province. The experimental animals were kept in cages with 5 animals per cage in a room with a temperature of 20–25°C and 50%–65% humidity with a light-dark cycle of 12 h light and 12 h dark. The rats were fed with food (rat pellet) and bottled water ad libitum. The experiments started after one week of acclimation of the animals in the new environment. The rats were 3 months old, weighing 240 ± 20 g at the commencement of experimentation. The animal protocol for this study was approved by the Institutional Animal Care and Use Committee of the Three Gorges University with an approved protocol number of 2017010E.

### 2.2. Drugs and Reagents [11]

SSTF (68% purity) was extracted by the College of Pharmacy of Three Gorges University. Briefly, *Sedum sarmentosum* was extracted twice, eight times each, with ethanol reflux, and the extract was concentrated at 1 : 1 under a vacuum. After degreasing with petroleum ether, the petroleum ether was dried to adjust the concentration and pH of the solution. Sampling and elution were carried out by the combination of macroporous resin and column chromatography polyamide. The extract solution was first added to a macroreticular resin column for adsorption for 24 h and then eluted with a 6-fold bed volume of ethanol (10%, 30%, 50%, and 70% sequentially) at 2 ml/min. Each elution fraction was collected and then subjected to polyamide column chromatography for further purification. After decompression and concentration, the eluent was freeze-dried to obtain the total flavonoid powder of *Sedum sarmentosum*. The composition of the total flavonoids was analyzed by UV spectrophotometry. *Schistosoma* positive *Oncomelania snails* were purchased from the Hubei Institute of Schistosomiasis Control and Prevention. *Schistosoma* cercariae were obtained from the *Oncomelania hupensis* snails by crushing the snails. Briefly, the bulk infected snail tissue was crushed and put into an Erlenmeyer flask, and then dechlorinated water (15–20°C) was added for 15 minutes. According to the distribution in the tail water, the cercariae were mainly floating on the water surface. They were collected by an inoculating loop under a microscope and immediately used to infect animals; Colchicine was purchased from the National Pharma Group Chemical Reagent Co., Ltd. (lot number of 20140226).

### 2.3. Grouping and Procedures

Sixty healthy male rats were randomly divided into 6 groups (10 in each group): normal control group; infected groups without treatment (model control group); low-dose SSTF group (100 mg·kg^−1^); medium-dose SSTF group (200 mg·kg^−1^); high-dose SSTF group (400 mg·kg^−1^); and colchicine positive drug control group (0.1 mg·kg^−1^). Except for the normal control group, the other groups were used to investigate schistosomiasis-induced hepatic fibrosis as described below.

**Figure 1:**
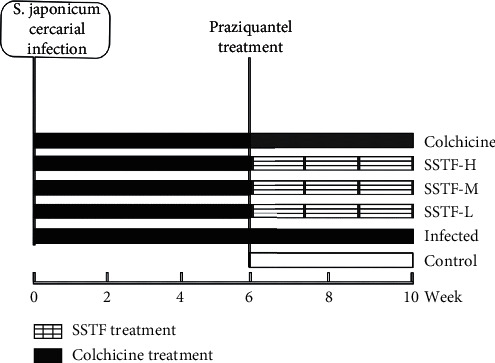
Experimental design with *S. japonicum* infection and treatment schedule.

The hepatic fibrosis model with schistosomiasis japonica was established as follows. Fifty male rats were infected with *Schistosoma japonicum* cercariae (40 ± 5) using the abdominal wall plucking patch method. The rats were given a routine diet, the rat pellets (carbohydrate 60%, protein 22%, fat 10%, other 8%). According to the above groupings, SSTF and colchicine were administered once a day by gavage, starting after 6 weeks of infection for 4 weeks along with simultaneous PZQ treatment for 2 days (40 mg·kg^−1^). The general condition, including mental state (good, wilting, general, or normal), appetite (food consumption per day), weekly weight gain, and bowel movements (feces status), of infected rats, was analyzed in all groups to evaluate drug effect. At the end of the 10^th^ week, the rats were fasted and euthanized under anesthesia with sodium pentobarbital. A part of the right lobe of the liver tissue was harvested for further experiments (Figure 1).

### 2.4. Light Microscopy Analysis for Morphological Changes of Liver Tissue

The liver was first examined for its texture and color and adhesion to its surrounding tissues. Then, approximately 1 g of liver tissue was fixed in buffered formalin (4.0% solution) for 24 h. After embedding in paraffin, the tissues were cut into 4 *μ*m thick sections, and dewaxed with xylene and then exchanged to ethanol. The tissue sections were then rehydrated in gradient ethanol to water and followed by heat-induced antigen repair solution (Maixin, Fujian, China). The slides were stained with the Masson staining kit (Solarbio, Beijing, China) following the manufacturer's instructions: (1) Weigert's iron hematoxylin staining for 5 minutes, followed by tap water rinsing, and then 1% hydrochloric acid alcohol differentiation for several seconds and tap water rinsing for several minutes; (2) Ponceau staining for 5–10 minutes followed by rinsing with distilled water; (3) Phosphomolybdic acid aqueous solution treatment for approximately 3–5 minutes; (4) Aniline blue solution for 5 minutes; (5) 1% glacial acetic acid for 1 minute; (6) Dehydrated seal. At least five fields in liver slices of each rat were examined using light microscopy, image acquisition, and analysis. The degree of fibrosis was divided into four levels: “−”: no obvious collagen fibrosis; “+”: collagen fibers increased significantly, extending radiantly from the portal area or central vein without fibrous septum formation; “++”: collagen fibers increased significantly and formed a discontinuous fibrous septum; “+++”: collagen fibers were connected into a complete fibrous septum, dividing the liver parenchyma [14]. Five fields of view were randomly selected under a high power microscope of each slice, and the percentage of collagen area with the blue color in the total field area (positive area%) was calculated, and the average value was taken by using the Image J system software for statistical analysis of pictures.

### 2.5. Immunohistochemistry Analysis of TGF-*β*1 Protein Expression in Liver Tissue

Immune staining of the liver tissue slides was performed following the kit instructions. (Maixin, Fuzhou, China). Briefly, the liver tissues were fixed with 4% paraformaldehyde, then paraffin-embedded, sectioned, dewaxed, and rehydrated. After blocking the peroxidase with 3% H_2_O_2_ methanol, antigens were retrieved by autoclave. After blocking with 5% bovine serum albumin (BSA) serum for 10 minutes, the slides were incubated with rabbit anti-rat TGF-*β*1 monoclonal antibody (1 : 500, Santa Cruz, USA) overnight at 4°C. On the next day, after rinsing three times with phosphate-buffered saline (PBS), the slides were incubated with the HRP-conjugated secondary antibody (sheep anti-rabbit, 1 : 200, Boster, Wuhan, China). After rinsing, the slides were stained in 1% DAB solution (Boster, Wuhan, China). The slides were restained with hematoxylin, followed by dehydration, and mounted with coverslips. Slides were washed in PBS three times for 5 minutes each time between two steps. For light microscopic examination, five observation fields (avoiding edge effect) were selected at the four corners and the central part of the section. Each observation field consisted of one portal area plus one hepatic lobule or a fibrous interval. The number of TGF-*β*1 positive cells was counted.

### 2.6. Western Blot Analysis of TGF-*β*1 and Smad7 Protein Expression in Liver Tissues

Liver tissue (1 mg) was placed in a microcentrifuge tube containing RIPA Lysis buffer (Applygen, Beijing, China) and homogenized on ice. After centrifugation, the supernatant was transferred to a new tube, and protein concentration was determined using the BCA Protein Assay (Applygen, Beijing, China). After lysing, 18 *μ*g of protein was loaded onto and resolved by SDS-PAGE electrophoresis (Bio-Rad, USA) and electrotransferred onto a polyvinylidene difluoride (PVDF) membrane (Millipore, USA). The membranes were blocked with 5% nonfat milk in PBS-Tween 20 (PBS-T) for 2 hours at room temperature, and the membranes were incubated overnight with the specific primary antibodies against TGF-*β*1 (rabbit anti-rat TGF-*β*1 monoclonal antibody, 1 : 1,000, AbCam, UK), Smad7 (rabbit anti-rat, Smad7 monoclonal antibody 1 : 500, Sigma, USA), or GAPDH (rabbit anti-rat GAPDH monoclonal antibody 1 : 2,000, Beyotime, China), respectively. On the next day, the membranes were washed with PBS-T three times and then incubated with sheep anti-rabbit secondary antibody conjugated with HRP (Antgene, Wuhan, China) for 1 hour at room temperature. The specific protein bands were detected with chemiluminescence in a darkroom using BeyoECL Plus (ECL Chemiluminescence Kit, ASPEN, WuHan, China) and documented and analyzed using Image Acquisition and Analysis Software (Flour Chem FC3 software, Alpha Innotech, USA).

### 2.7. RT-qPCR Quantitative Analysis of TGF-*β*1 and Smad7 mRNA Levels in Liver Tissues

Total RNA in the liver tissue was extracted using RNAiso Plus (D9108A, Takara, Da Lian, China). RNA purity and concentration were analyzed using UV spectrophotometry (A260 : A280 > 1.8 was considered satisfactory). Total RNA (2 *μ*l at a concentration of 0.2 *μ*g/*μ*l for each sample) was reverse-transcribed into cDNAs using the PrimeScript RT reagent kit (RR047A, Takara, Da Lian, China). At the end of the RT reaction, RNase-free H_2_O was added up to 50 *μ*l, and the sample was then stored at −20°C for use in the next step. The cDNAs (13.6 *μ*l) were used for qPCR using Roche LightCycler 480 (Switzerland) in a 96-well plate with SYBR Premix Ex Taq™ kit (DRR041A, Takara, Da Lian, China). The thermocycling parameters were 95°C for 10 seconds; 40 cycles of 95°C for 5 seconds and 60°C for 20 seconds; and 65°C for 15 seconds. GAPDH was used as an internal control. All primer sequences are listed in Table 1. The results were analyzed and quantified using the Roche LightCycler 480 software. Target gene expression (2^−ΔΔCt^) was normalized to the endogenous GAPDH mRNA expression. The amount of the target gene in the control sample was set at 1.0. Thus, the resulting gene expression relative to GAPDH was further normalized to the average of the target gene in the normal control group.

### 2.8. Statistical Analysis

All data analyses were performed with the SPSS19 software (version 19.0; SPSS, Inc., Chicago, IL, United States), and all data are presented as the mean ± standard deviation (SD). One-way analysis of variance (ANOVA) was used to compare the difference between 6 groups. A *P* value <0.05 was considered statistically significant.

## 3. Results

### 3.1. SSTF Treatment Improves the General Condition of Infected Rats without Affecting Survival

**Figure 2:**
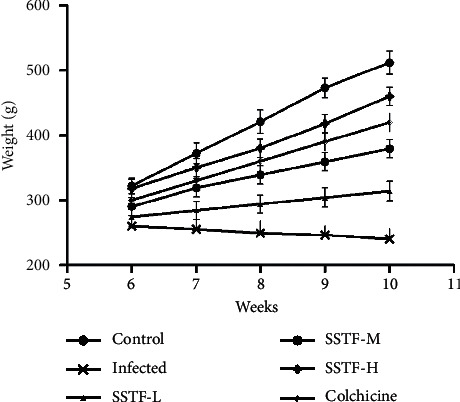
Weekly changes in body weight for all 6 groups over the duration of treatment. Weight comparisons between the 6 groups were significantly different (ANOVA *P* < 0.05) by the end of 10^th^ week. There was no significant difference between the infected group and the SSTF-L group; between the SSTF-L, SSTF-M, and colchicine groups; between the SSTF-M, SSTF-H, and colchicine groups, and between the SSTF-H, control, and colchicine (*P* > 0.05).

Rats in the normal group had a good mental state, good appetite, normal weekly weight gain, normal bowel movements, and no death at the end of the experiment. In the infected group, body weight either did not increase or slowly decreased with time, and the spirit was wilting, activity and appetite decreased, stool did not form, urine was yellowish, and one rat died at the end of the experiment. The SSTF low-dose group was generally better compared to the infected group. Specifically, weight gain slowed and the appearance of the rats was similar to the normal group and the medium and high-dose SSTF groups. One rat died in the middle dose group, and two rats died in the low-dose group. The colchicine positive group showed similar performance to the SSTF-intervention group; one animal died at the end of the experiment. Weekly body weight changes during treatment are shown in Figure 2. Compared to the normal control group, the infected group without treatment exhibited a slow decrease in body weight over time. Notably, SSTF dose-dependently increased body weight compared to the infected group and similar to the normal control (Table 2).

### 3.2. SSTF Treatment Decreases Hepatic Fibrosis in Infected Rats

**Figure 3:**
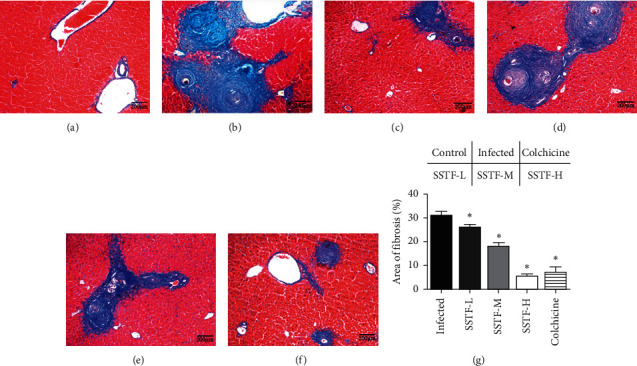
Masson staining of the effect of SSTF on liver morphology. Representative images of liver tissues in 6 groups: (a), normal control, (b) infected, (c) colchicine, (d) SSTF-low dose, (e) SSTF-medium dose, (f) SSTF-high dose. Blue staining represents collagen relative to the degree of fibrosis. Scale bar: 200 *μ*m. (g) Quantitative results of images of the relative areas of fibrosis. Compared with the infected group, proliferation in the fibrous connective tissue decreased with increasing doses of SSTF (^*∗*^*P* < 0.05 compared to the infected group).

Compared with the control group, the other five groups of the liver showed obvious dark blue collagen deposition in the liver under Masson staining, indicating that the model of schistosome-induced liver fibrosis was successfully constructed. Liver lobules in the normal control group were well organized with the hepatocytes arranged radially around the central vein and a small amount of neatly arranged collagen fibers. In the infected group, the hepatic lobules were destroyed, with increased fibrous tissue, cell proliferation, and collagen deposition (blue color in Masson trichrome staining) observed in the central vein, portal area, and hepatic parenchyma. The area of fibrosis was approximately 30%. SSTF treatment significantly reduced the area of fibrosis in a dose-dependent manner (Figures 3(a)–3(g)). The SSTF high dose group exhibited the highest reduction (6-fold) of fibrosis, and the extent of the reduction was similar to that in the colchicine positive control group. Thus, SSTF treatment can significantly alleviate hepatic fibrosis induced by schistosomiasis.

### 3.3. SSTF Treatment Alters Liver TGF-*β*1 and Smad7 Protein Expression in Rats with Hepatic Fibrosis

**Figure 4:**
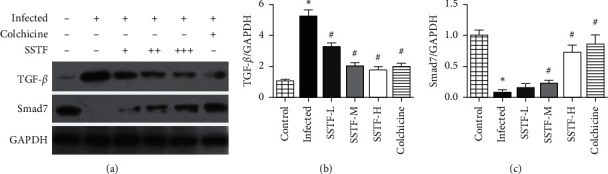
SSTS effects on TGF-*β*1 and Smad7 protein expression in liver tissues of rats with hepatic fibrosis. (a) Western blot results of TGF-*β*1, Smad7, and GAPDH in 6 groups. (b) Quantitative result of TGF-*β*1 normalized to GAPDH. (c) Quantitative result of Smad7 normalized to GAPDH (^*∗*^*P* < 0.05 compared to the normal control group; ^#^*P* < 0.01 compared to the infected group).

To explore the mechanism of SSTF's effect on liver fibrosis, we measured TGF-*β*1 and Smad7 protein expression.  Figure 4 shows the Western blot results of TGF-*β*1 and Smad7 in the liver samples. Compared with the normal control group, TGF-*β*1 protein expression in the infected group was significantly increased (by ∼5 fold), whereas Smad7 protein expression was significantly decreased by ∼10-fold (*P* < 0.01). Compared with the infected group, SSTF treatment significantly and dose-dependently reduced TGF-*β*1 protein expression, with the highest level of reduction similar to that in the colchicine group. SSTF treatment also normalized Smad7 protein expression in the infected liver tissue, with the greatest increase observed in the high-dose group similar to that of the colchicine positive control group. Thus, SSTF treatment can significantly prevent an increase in TGF-*β*1 and a decrease in Smad7 protein expression in the liver tissue following schistosomiasis infection.

### 3.4. Liver TGF-*β*1 and Smad7 mRNA Levels in Rats with Hepatic Fibrosis

**Figure 5:**
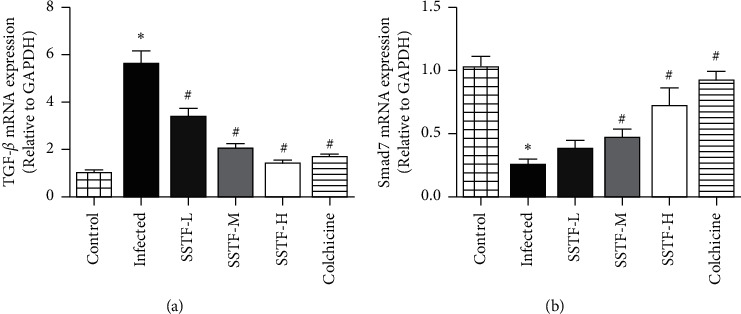
SSTF effects on TGF-*β*1 and Smad7 mRNA levels in liver tissues of rats with hepatic fibrosis. qPCR results first normalized to the internal control GAPDH and then renormalized to the mean of the normal control level. (a) TGF-*β*1 mRNA levels relative to normal control. (b) Smad7 mRNA levels relative to normal control (^*∗*^*P* < 0.05 compared to the infected group; ^#^*P* < 0.01 compared to the infected group).

Changes in protein expression can be the result of alterations in protein production or degradation. Thus, we further measured TGF-*β*1 and Smad7 mRNA levels. Compared with the normal control group, TGF-*β*1 mRNA levels in the liver tissue of the infected group were significantly increased, whereas Smad7 mRNA levels were significantly decreased (*P* < 0.01; Figure 5). SSTF treatment significantly and dose-dependently normalized these pathological changes of TGF-*β*1 and Smad7 mRNA levels, with the greatest effects being similar to that in the colchicine positive control. The effects of SSTF on TGF-*β*1 and Smad7 mRNA levels paralleled the effect of SSTF on protein expression, suggesting that SSTF primarily alters protein synthesis of TGF-*β*1 and Smad7, although we cannot completely exclude the possibility that SSTF influences protein degradation.

### 3.5. Immunohistochemistry Analysis of TGF-*β*1 Expression in Hepatocytes

**Figure 6:**
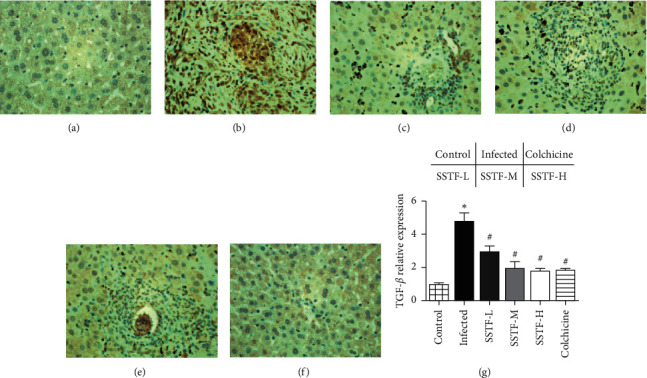
Immunostaining of TGF-*β*1 in liver tissues (×40) of six groups: (a) normal control, (b) infected, (c) colchicine, (d) SSTF-low dose, (e) SSTF-medium dose, and (f) SSTF-high dose. Scale bar: 100 *μ*m. Optical densities of the images were analyzed using IPP software. (g) After *schistosome* infection, the rate of TGF-*β*1 positivity significantly increased (^*∗*^*P* < 0.05 compared to the normal control group) in the infected group, and SSTF treatment markedly decreased the rate of TGF-*β*1 positivity (^*∗*^*P* < 0.05 compared to the control group; ^#^*P* < 0.01 compared to the infected group).

To visualize TGF-*β*1 expression in the liver, we also performed immunohistochemical staining.  Figure 6 shows that there were no TGF-*β*1 positive cells in normal liver tissues. In the infected group, however, a large number of TGF-*β*1 positive cells were observed around the portal area and central vein, with a small number of hepatocytes observed around the fibrous septum. In addition, there were numerous cells with deep staining or with a wide range of positive staining. SSTF treatment progressively decreased the TGF-*β*1 positive staining and reached a level of reduction similar to that in the positive treatment control group with a high dose of SSTF.

## 4. Discussion

Schistosomiasis is a major public health concern worldwide. Hepatic fibrosis from two major subtypes of Schistosomiasis, *S. Mansoni* and *S. japonicum*, is directly related to morbidity and mortality. However, there is currently no effective treatment for hepatic fibrosis. To this end, we explored a new treatment of hepatic fibrosis using an extract of Chinese herbal medicine, SSTF, which has been reported to reduce fibrosis following liver and kidney injury [13]. Our results demonstrate that SSTF can significantly and dose-dependently reduce *S. japonicum*-induced liver fibrosis. We also observed the impact of SSTF on key signaling molecules in fibrosis formation. Our results revealed that SSTF treatment could significantly reduce the expression of the profibrotic molecule TGF-*β*1 in the liver following *S. japonicum* infection at both the mRNA and protein levels. In contrast, SSTF treatment reversed the reduction of the antifibrotic molecule Smad7. While a detailed mechanism remains to be elucidated, our study provides a potential molecular mechanism of SSTF's antifibrotic effect.

Hepatic fibrosis is a chronic, progressive, and diffused pathological change of the liver caused by many factors. It is characterized by HSC proliferation and ECM deposition. Hepatocyte degeneration and necrosis after liver injury are followed by regeneration and fibrous tissue proliferation. Severe liver cirrhosis can often result in hepatocellular carcinoma [15, 16].

TGF-*β*1 is mainly produced by hepatic parenchymal cells and HSCs in the liver. Its expression can accurately determine the degree of hepatic fibrosis. TGF-*β*1 is one of the most potent hepatic fibrosis promoting factors and is involved in nearly all of the key aspects of liver fibrosis [17]. TGF-*β*1 generally promotes fibrosis through the following three mechanisms [18, 19]: (1) inhibiting ECM degradation by promoting the expression of tissue inhibitor of metalloproteinases (TIMPs) and inhibiting the expression of matrix metalloproteinases (MMPs); (2) inducing the formation of myofibroblasts by epithelial-mesenchymal transition (EMT); (3) promoting HSC activation and formation of fibrosis through the Smads-dependent pathway and other non-Smads pathways, such as MAPK, NF-*κ* B, PI3K, and others. Thus, the inhibitory effect of SSTF on liver TGF-*β*1 expression following *S. japonicum*-induced infection in our study suggests that one main mechanism of SSTF's antifibrotic effects is through controlling the expression of Smads.

It is currently believed that the Smads protein is a critical negative regulator of TGF-*β*1 signaling transduction from the cell membrane receptor to the nucleus. Smad7 can reverse TGF-*β*1 signaling to achieve antifibrotic effects [19]. Overexpression of Smad7 inhibits the phosphorylation of the TGF-*β*1 receptor and prevents activation of TGF-*β*1 signaling. By reversing the TGF-*β*1/Smads signaling pathway, Smad7 can reduce the expression of type I collagen and *α*-smooth muscle actin in activated HSCs. Thus, Smad7 plays an important anti-fibrosis role [17, 18, 20]. The TGF-*β*1/Smads signaling pathway is the most classical pathway of TGF-*β*1 in promoting fibrosis. It plays a significant role in HSC activation and proliferation, ECM deposition, and hepatic fibrosis.

Currently, there is no ideal antifibrotic drug to treat hepatic fibrosis [21]. Previous studies have shown that some Chinese medicines can reverse hepatic fibrosis by reducing TGF-*β*1 and increasing Smad7. For example, silymarin and oxymatrine can significantly reduce TGF-*β*1 expression in rat models of hepatic fibrosis [22, 23]. Shikonin and astragaloside can downregulate Smad3,4 and upregulate Smad7 expression [6, 24]. Thus, the effect of traditional Chinese medicine on TGF-*β*1 expression and the reversal of hepatic fibrosis have become a current focus. Although SSTF has been reported to prevent hepatic fibrosis in other hepatic injury models to some extent due to its anti-inflammatory properties, the mechanism remains unclear. SSTF is the main active ingredient extracted from *Sedum sarmentosum*. Our study provides further insights into the molecular mechanism of SSTF's antifibrotic effects by inhibiting the TGF-*β*1/Smad pathway. Our results suggest that SSTF can not only inhibit the expression of a key profibrotic molecule but can also promote the expression of a key antifibrotic molecule in the same signaling pathway. This dual action of controlling the expression of key signaling molecules may explain SSTF's strong antifibrosis effect.

The SSTF used in this study is a complex plant extract containing many components. The biological activity of SSTF could be a synergistic effect of its multiple active components. Alternatively, it may contain only a single active component. The detailed molecular mechanism of SSTF's effect on alleviating schistosomiasis japonica-induced hepatic fibrosis needs to be further elucidated. In addition, we observed that even though SSTF can significantly alleviate hepatic fibrosis, it did not improve survival in our rat model. A similar phenomenon was also observed for the colchicine positive control. Thus, the death of the infected animals in our experimental groups may not be directly related to hepatic fibrosis and could be the result of acute systemic hypersensitivity, warranting further investigation.

Our study shows that SSTF treatment can reduce schistosomiasis-induced liver fibrosis by downregulating TGF-*β*1 and upregulating the Smad7 expression in liver tissue, thus preventing TGF-*β*1 from activating HSCs and reducing the degree of hepatic fibrosis. Our study demonstrated that SSTF has the potential to be developed as an antifibrotic drug. Further research is needed to determine whether the effects of SSTF on TGF-*β*1 and Smad7 are direct effects or indirect effects through acting on other molecular target(s) and whether other pathways are also affected by SSTF treatment.

## Figures and Tables

**Table 1:** Primers used in RT-qPCR.

Gene	Primer sequences (5′-3′)	
*TGF-β1*	Forward Reverse	AGGCGGTGCTCGCTTTGTA GATTGCGTTGTTGCGGTCC

*Smad7*	Forward Reverse	TACCTTCCTCCGATGAAACCG GAGTCTTCTCCTCCCAGTATGCC

*GAPDH*	Forward Reverse	AAGTTCAACGGCAGTCAAGG CGCCAGTAGACTCCACGACATA

**Table 2:** General conditions of 6 groups of rats ($\bar{\chi }\pm s$).

Group	*n*	Weight gain (g)	Food consumption/day (g)	Death (*n*)	Mental state	Stool traits
Normal	10	243.1 ± 8.2	32.8 ± 0.8	0	Good	Normal
Infected	10	−(84.5 ± 13.4)^*∗*^	18.6 ± 0.9^*∗*^	1	Wilting	Not form
Low-dose SSTF	10	52.7 ± 12.5^*∗*^	20.3 ± 0.6^*∗*^	2	Wilting	Not form
Medium-dose SSTF	10	83.1 ± 15.3^*∗*^	21.8 ± 0.7^*∗*^	1	General	Not form
High-dose SSTF	10	140.8 ± 13.8^*∗*^	22.9 ± 1.1^*∗*^	0	Normal	Form
Colchicine positive	10	138.1 ± 19.2^*∗*^	23.1 ± 0.9^*∗*^	1	Normal	Form

Note: compared with group normal, ^*∗*^*P* < 0.05.

## Data Availability

The datasets generated and analyzed during the present study are available from the corresponding author upon reasonable request.

## Abbreviations

SSTF: *Sedum sarmentosum* total flavonoids
TGF-*β*1: Growth factor-beta 1
PZQ: Praziquantel
BSA: Bovine serum albumin
ECM: Extracellular matrix
qRT-PCR: Quantitative real-time PCR
HSC: Hepatic stellate cell
PVDF: Polyvinylidene difluoride
SD: Sprague-Dawley
TIMPs: Metalloproteinases
MMPs: Matrix metalloproteinases
EMT: Epithelial-mesenchymal transition
ANOVA: One-way analysis of variance.
